# Supplementary material for: Tracing social mechanisms and interregional connections in Early Bronze Age Societies in Lower Austria
Source: Nat Commun. 2025 Dec 31;17:131. doi: 10.1038/s41467-025-67906-y (PMC12775072; doi:10.1038/s41467-025-67906-y)
Supplement: Supplementary file 1 — Supplementary Information [file 41467_2025_67906_MOESM1_ESM.pdf]

Supplementary Information: “Tracing social mechanisms and interregional connections in Early Bronze Age Societies in Lower Austria”

Supplementary Note 1: Archaeological Information on the sites

Drasenhofen

Archaeological excavations on both sides of the Stützenhofner Bach Creek were initiated in 2018 in response to the construction of a motorway bypass for Drasenhofen at the Austrian-Czech border[1]. The Early Bronze Age site includes extensive settlement remains, dating to the classical Únětice culture and early Věteřov culture (c. 2150-1700 BCE). Four isolated buildings, consisting of two workshops and two dwellings, were found at the southeastern end of the excavation area, forming a small farmstead separated from the larger, village-like settlement[1]. The cemetery was located south of the village and north of the farmstead, with 25 burials in 22 graves arranged in four rows potentially representing former inhabitants of the settlement[2,3]. The topography of the site suggests that all the graves of the cemetery were located within the construction line and the grave group was completely excavated. The cemetery's block-like, regularly rectangular shape of approximately 30 × 50 m implies that its boundaries were likely demarcated, for example by a fence. Outside the cemetery, four additional individuals were discovered buried in former storage pits within the settlement area. With 15 female and 14 male individuals, the sex ratio is balanced; eleven individuals died before they reached the age of 20. In the course of our investigation, we found the oldest genetic evidence of two different strains of the plague bacteria (Yersinia pestis) in male individuals, who died at the ages 23-30 and 22-27 years of age, respectively. To date, this is the earliest evidence of Late Neolithic / Early Bronze Age plague in today’s Austria[4].

Zwingendorf

In 1977, the archaeological site of Zwingendorf, Austria, yielded a total of nine Early Bronze Age graves during excavation in 1977[5,6]. A radiocarbon sample from Zwingendorf (NHMW-ANTHRO-OSTE-1008585/8A) was sent to the Higham laboratory at the University of Vienna and measured at the Vienna Environmental Research Accelerator AMS facility. The results ranged from 2120 to 1945 cal. BCE

confirmed the typo-chronological dating that has placed the site within the developed Únětice culture, estimated from approximately 2000 to 1600 BCE (Supplementary Figure 1). The small grave group with north-south orientated grave pits revealed ten remarkably undisturbed individuals interred in flexed positions, all on the right sides of their bodies with their heads towards the south. Grave 8 included a 4-year-old child with a bronze ring placed in front of a 15-16-year-old adolescent's body. The grave further contained five ceramic vessels.

Unterhautzenthal

Unterhautzenthal, an Early Bronze Age site of the Únětice culture, comprises both remnants of a settlement, including storage pits in which human remains have been found, and a small cemetery excavated in the 1980-90s[7,8,9]. Recent re-analysis of the human remains from Unterhautzenthal has provided evidence of additional individuals, primarily neonates and children, who were not individually documented at the time[10]. The inclusion of these skeletons has expanded the total count of excavated individuals at Unterhautzenthal to fifty-eight, with thirty-two (55%) representing sub-adults under the age of twenty. Almost all bodies were placed in a flexed position on the right side of the body; one was found in a prone position. The predominant body orientation was with the head to the southwest, south, and southeast, with a few exceptional cases oriented to the north. The triple burial 95 contained the remains of a 35-45-year-old woman and two children, who died at the ages of 2-3 and 4-5 and were placed in close contact with the woman's body. The burial of two closely related children in a storage pit, a 2 and a 6-year-old in an embrace, is further noteworthy[11].

Schleinbach

Schleinbach, situated 10 km northeast of Vienna in Lower Austria, lies north of the Danube and represents a cemetery and settlement complex associated with the Únětice culture. The Bronze Age settlement, encompassing approximately 1.5 hectares, likely neighbored a prehistoric lake. Initial discoveries were made in 1911 within a brick factory, and more or less systematic excavations accompanied the clay extraction until the 1980s[12,13]. Within the site, two distinct groups of Early Bronze Age burials were uncovered: Group 1 located in the western area, and Group 2 in the eastern area of the site, at approximately 180 m distance. The western grave group

with graves arranged in two rows included the double burial of two male individuals 30/31 with identical head fractures[14]. Storage pits with human remains, such as the quadruple burial of an adult male and three children in Pit 60[14] and the burial of a 5-6-year-old boy with no fewer than four fatal blunt force traumata to the skull in Pit 3/1981[12] contribute to the richness of the site's historical record and its recognition within Austrian prehistory. The eastern grave group included 17 burials, many of which were heavily disturbed, with few grave goods and intermingled human remains. The skeletal remains of 62 individuals were available for a recent osteological re-analysis[15], which found a demographic composition of 14 adult and mature females, 15 adult and mature males and 27 subadults, some of which were fetuses and neonates represented only by a few bones. The study further found extraordinary levels of stress indicators and traumas at the site, testifying to violence, conflict, abuse and marginalization at Schleinbach.

Ulrichskirchen

Ulrichskirchen is an Únětice culture site complex excavated in 2011 in advance of pipeline construction. It includes settlement features with human remains deposited in pits, as well as a group of formal graves. The Schleinbach and Ulrichskirchen sites are located only about 1 km apart, and can thus be interpreted as components of a larger, contemporary complex.

The first archaeological intervention uncovered a small cemetery with 11 individuals, as well as five additional individuals deposited in former storage pits. These finds are currently being prepared for publication by Maria Teschler-Nicola and Friederike Novotny. A second intervention led to the discovery of four more individuals buried in storage pits and one individual in a grave, all of whom were the focus of Domnika Verdianu’s Master’s thesis[16].

The graves were arranged in two rows of five and one additional row of two graves. However, since the excavation area was limited to the width of the pipeline, further graves may be expected. All burials were aligned along a northeast–southwest axis and contained single individuals, laid on their right side in a flexed position with the head facing south. All graves showed signs of having been reopened at some point after burial.

One settlement pit contained four individuals, another two, while the rest each contained a single individual. Whereas the multiple depositions suggest that the bodies may have been discarded, two of the single burials appear to have been carefully placed, one of them even oriented in accordance with prevailing funerary customs.

Franzhausen

The cemeteries of Franzhausen I and II, situated in the Traisen Valley of Lower Austria, are part of an expansive archaeological landscape revealed by large-scale rescue excavations in the last decades of the 20th century. The ca. 2200 burials span the entire period of the Early Bronze Age from about 2300–1600 BCE (Supplementary Figure 1) and are associated with the Unterwölbling cultural group[17]. The dead were buried in flexed, gender-specific body positions, usually in individual graves. Women were placed on the right side of the body, head south, whereas men were placed on the left, head north. Grave goods comprise bronze costume and jewelry, weapons and tools, ceramic vessels as well as meat products. Merely twelve of the 716 excavated graves at Franzhausen I contained double or multiple burials[18]. The triple burial 599 is one of these exceptional cases, where the remains of a 20–25-year-old male individual (designated as 599A) were found positioned on the left side with the head oriented to the north-west in a wooden coffin. The upper part of the man's body was severely disturbed and his cranium was missing. Two adolescent individuals, 14–16 (designated as 599B) and 12–14 (designated as 599C) years at the time of death, accompanied the adult male. Their bodies were positioned at the adult’s feet in the south-eastern part of the coffin, on their left sides with their heads to the south-east. The lower legs of the young individuals came into close contact, suggestive of a deliberate placement. The burial sequence indicates that the adult male was laid in the coffin first, followed by the older adolescent, and finally, the youngest was positioned behind the older one's back in a single act of deposition[19].

Pottenbrunn

The site of Pottenbrunn[20,21] is situated in Lower Austria south of the Danube in the Traisen Valley, approximately 12 km south of the more famous sites Franzhausen I and II. The site was excavated in 1981/82 in the course of rescue excavations, following the same digging and recording system as for Franzhausen. Pottenbrunn

belonging to the Unterwölbling culture seemed to have been completely excavated and includes Early Bronze Age settlement structures, with graves placed in abandoned areas of the settlement, and a total of 74 roughly north-south oriented grave pits. Of those, 69 included human remains. All were single graves, but four graves included remains of more than one individual[20]. The site was heavily disturbed, not only by contemporaneous grave re-opening, but also by a La Tène cemetery built on top of the site several hundred years later[22]. The cemetery population includes 19 females and 20 males, as well as 37 subadult individuals[21]. In many ways, burial practices are comparable to Franzhausen I, with gendered burial placement, orientation, and grave goods, but overall poor preservation.

C14 dating of individuals of different sites/cultures

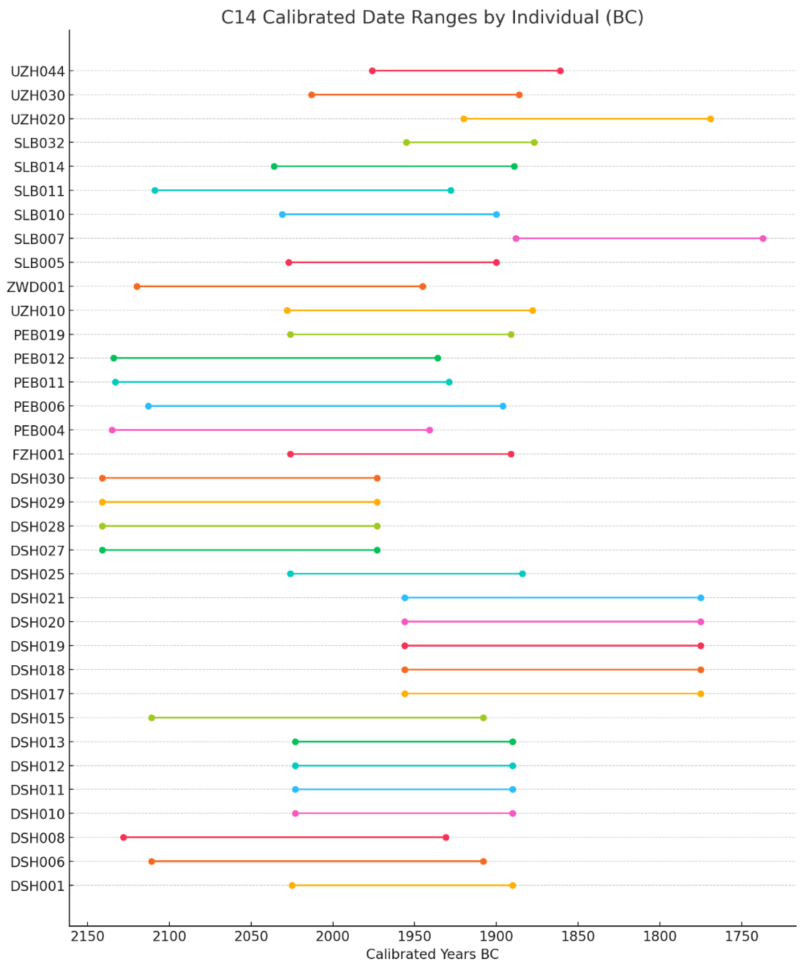

Supplementary Figure 1. Radiocarbon (^{14}C) Calibrated Date Ranges by Individual. Colored horizontal lines show independent ^{14}C determinations for each individual; the dots mark the calibrated range endpoints (95% probability). X-axis is calibrated years BC.

Supplementary Note 2: Evaluation of bias from different wet-lab methods on genetic kinship analysis

Of the 15 samples processed in Boston, 11 (I28636, I28642, I28991, I28992, I29242, I29247, I29687, I29691, I29693, I11700, I11704, I11705, I28635, I28641, I29246) were captured using a new enrichment method (Twist1.4M).

To ensure that combining these samples with those captured using the 1240k method would not introduce bias, we analyzed pairwise mismatch rates (Fig. 2). While there is a slight tendency for the 1240k samples to be more similar to each other than to the Twist samples, this bias is minimal and does not affect the relatedness outcomes in our relationship analysis.

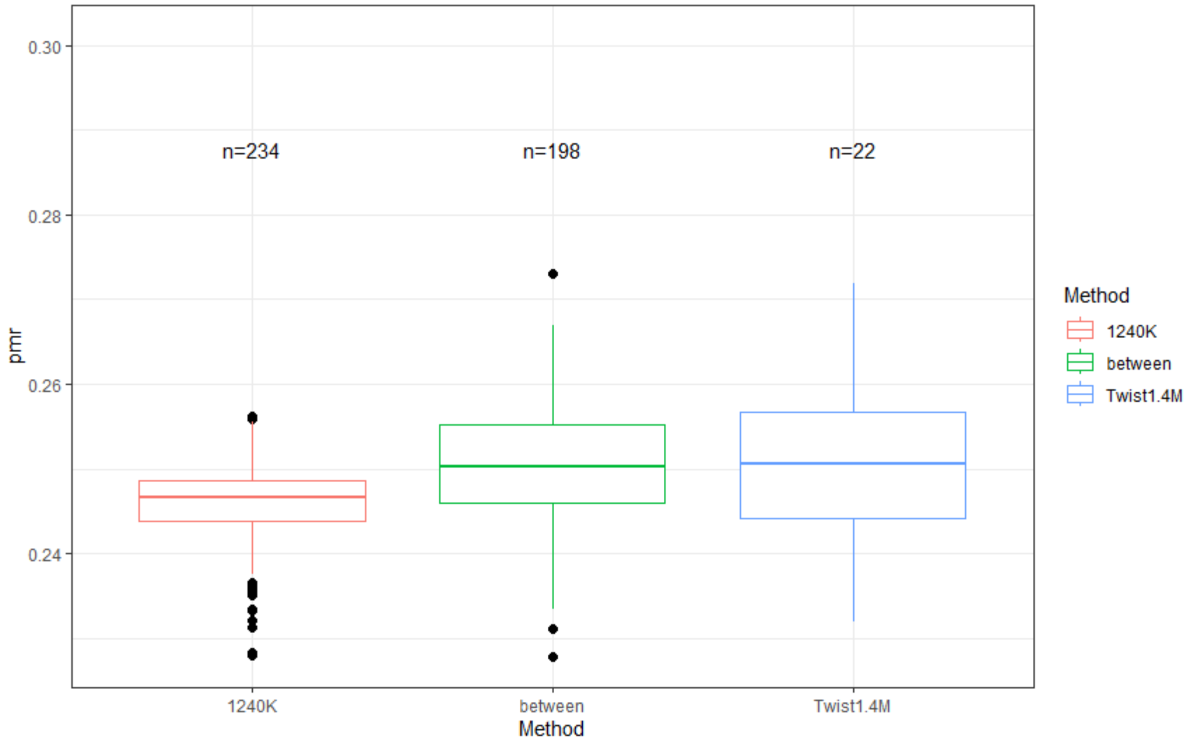

Supplementary Figure 2. Pairwise mismatch rates within 1240k and Twist samples (red and blue) and between the two groups (green). The comparison between individuals capture with the same and different capture methods shows minimal differences.

Supplementary Note 3: outgroup f3 statistics on populations used in IBD analysis

To complement the IBD (Identity-by-Descent) analysis, we conducted pairwise qpWave tests using Mbuti as an outgroup to evaluate the number of ancestral sources needed to explain genetic relationships between populations. The results are presented in Supplementary Figure 3, where higher p-values (blue) suggest that two populations can be modeled with a shared ancestry stream, indicating greater genetic similarity, while lower p-values (yellow) imply the need for additional ancestral sources, reflecting more distinct genetic histories. As outgroup populations, we used: Mbuti.DG, Russia_Ust_Ishim_HG_published., CHG, EHG, Iberia_ElMiron, Czech_Vestonice16, Russia_MA1_HG.SG, Israel_Natufian_published, Jordan_PPNB_published, Anatolia_N_published, Anatolia_N, WHG, Iran_GanjDareh_N, Russia_EBA_Yamnaya_Samara, and Morocco_LN.SG.

Differences in ranking compared to the IBD analysis are expected, as IBD captures individual-level recent shared ancestry, whereas qpWave assesses shared ancestry at the population level, often reflecting deeper and more complex demographic events such as admixture or population structure. The qpWave and IBD results show partial overlap, with groups like Unterwölbling, Unetice, and Bad Zurzach displaying both shared ancestry streams and recent genetic connections. While not identical, the concordance suggests that some population relationships persisted across both deep and recent timescales.

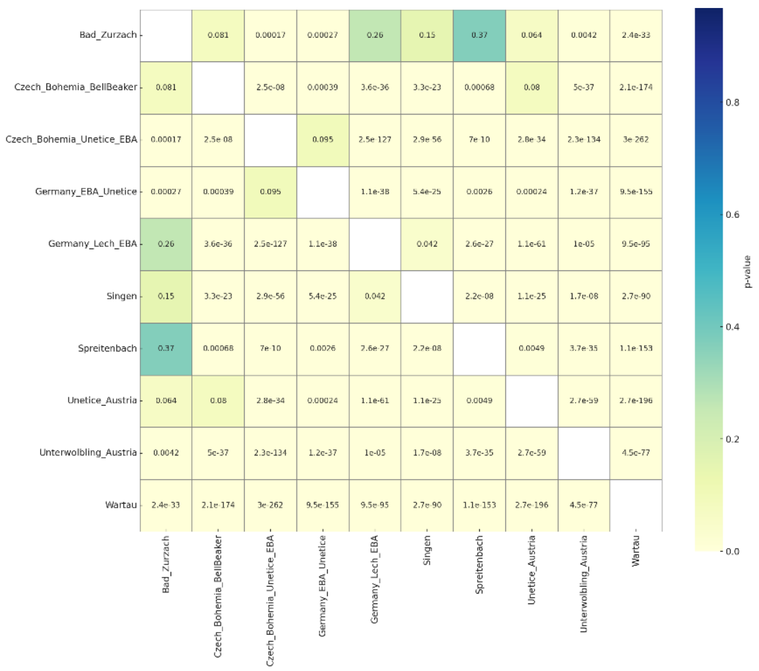

Supplementary Figure 3. **Pairwise qpWave heat map showing genetic similarities between populations.** Higher values (blue) indicate stronger shared genetic drift, while lower values (yellow) reflect less similarity. Sample sizes of the groups are the same as in main Figure 4.

Supplementary Note 4: Analysis of runs of homozygosity

We used the hapROH method (v.1.0) for the analysis of runs of homozygosity (ROH), which is specifically designed to analyze low-coverage aDNA data. By leveraging linkage disequilibrium from a panel of modern haplotype references, this method can successfully infer ROH longer than 4 cM on 1240K data with at least 0.3× coverage. In cases of close parental relatedness, which results in long ROH in offspring, hapROH efficiently detects very long ROHs even at lower coverage. We called ROH individuals with >250,000 SNPs. The program's embedded functions were used for plotting the ROH as combined histograms.

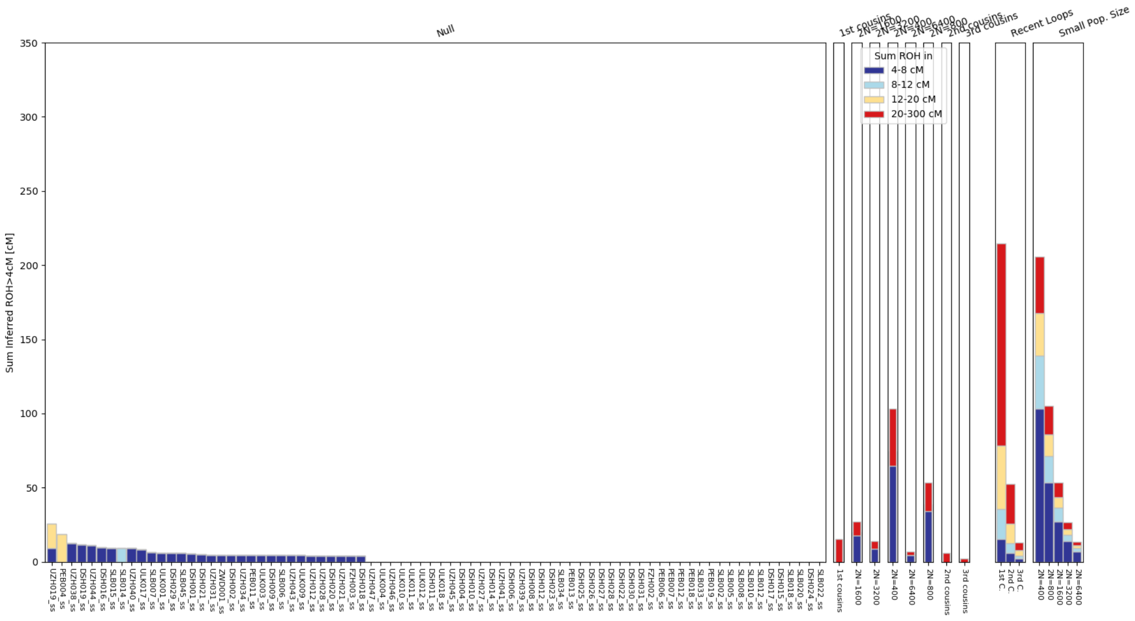

Supplementary Figure 4. Sum of runs of homozygosity (ROH) per individual. Left the individuals from the EBA sites in Lower Austria and right expected patterns of ROH for cousin-cousin marriage and small population size.

Supplementary Note 5: Reconstructed family trees per site

Drasenhofen

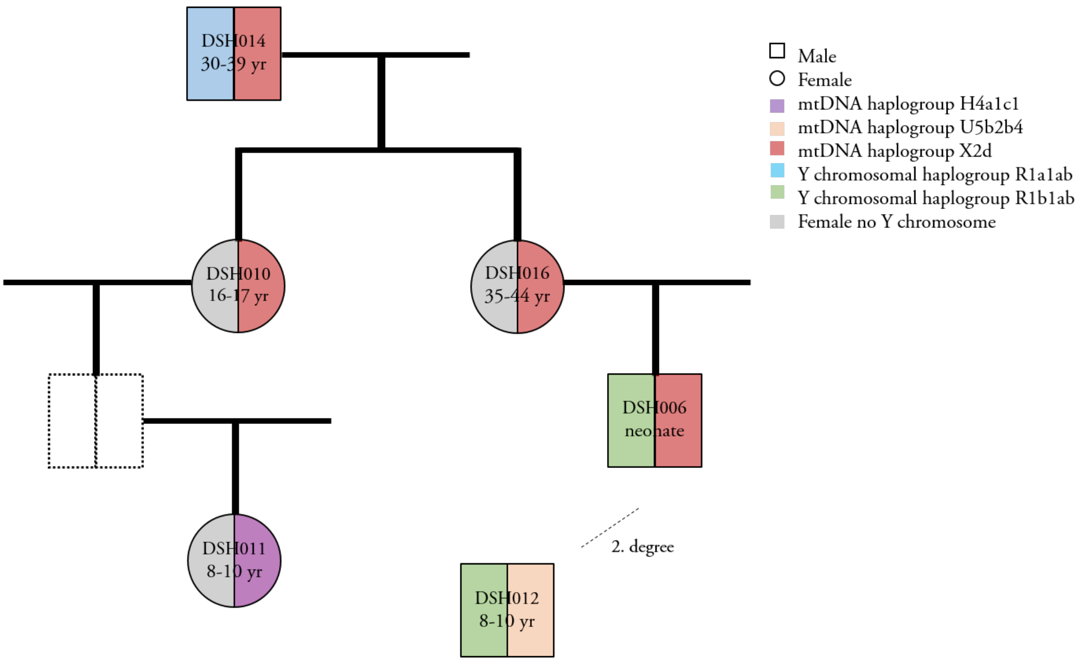

Supplementary Figure 5. Reconstructed family tree 1 Drasenhofen. This pedigree consists of six genetically analysed individuals (three males and three females): one adult male, one adult female and four subadults. We can reconstruct three consecutive generations, which are connected through both maternal and paternal lines. Pairwise mismatch rates identify DSH014, DSH010 and DSH016 as a trio of mutually first-degree relatives, and DSH016 as a first-degree relative of the neonate DSH006. DSH006 is second-degree related to DSH010, DSH011, DSH012 and DSH014, while DSH011 and DSH012 are second-degree relatives of several other family members. The pictured family tree shows the simplest way of arranging these relationships in a pedigree. However, some connections, especially those involving the second-degree relative DSH012 (and, to a lesser extent, DSH011), are not uniquely determined by the genetic data. The second-degree links between DSH006 and DSH012, and between DSH012 and DSH010/DSH016, could also plausibly reflect alternative configurations (e.g. paternal half-siblings, avuncular or grandparent–grandchild relationships). Thus, while the drawn pedigree is our most parsimonious reconstruction, other second-degree relationships cannot be ruled out.

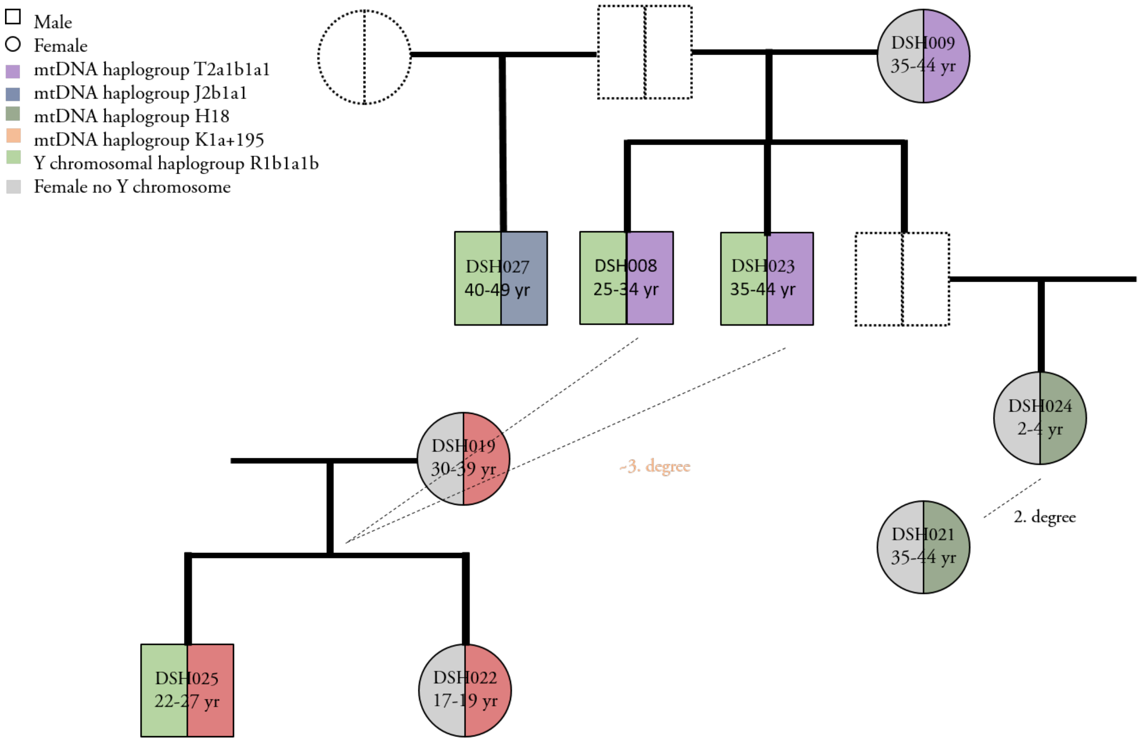

Supplementary Figure 6. Reconstructed family tree 2 Drasenhofen. This pedigree consists of nine individuals: four adult males, three adult females and two subadults. We can reconstruct four consecutive generations, which are connected through both maternal and paternal lines as indicated by shared mtDNA and Y-chromosomal haplogroups. The pictured family tree shows the simplest possibility of reconstructing the relationships in a pedigree. However, some connections are not 100 % clear: the inferred third-degree relationship involving DSH019 and the middle-generation males (DSH027, DSH008, DSH023) could also plausibly be realised through alternative configurations (for example different cousin or avuncular relationships). Likewise, the second-degree link between DSH021 and DSH024 could correspond to several genealogical scenarios such as a half-sibling, grandparent–grandchild or avuncular relationship. In addition to the relationships drawn in the pedigree, the pairwise mismatch rates show more distant kin ties between this family and the other reconstructed pedigree: DSH006 is approximately 4th-degree related to DSH008, DSH023 and DSH025; DSH012 is approximately 5th-degree related to DSH023 and DSH008; and DSH009 is approximately 6th–8th-degree related to DSH006, DSH014 and DSH016. In addition, the neonate DSH006 and the adult DSH027 form a possible first-degree pair linking (1077 SNPs) the two family groups.

Zwingendorf

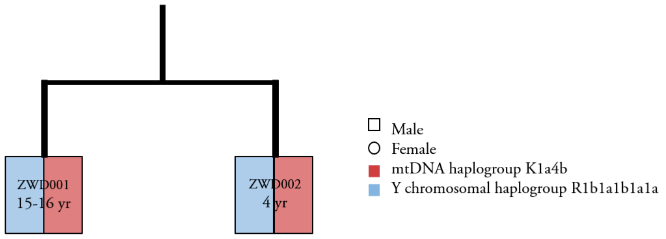

Supplementary Figure 7. Reconstructed family tree Zwingendorf. This pedigree from Zwingendorf (ZWD) consists of two genetically analysed individuals, both subadult males: ZWD001 (15–16 years at death) and ZWD002 (around 4 years). Both carry mtDNA haplogroup K1a4b and the same Y-chromosomal haplogroup R1b1a1b1a1a, indicating that they share both their maternal and paternal lineages. Together with their autosomal similarity, this points to a first-degree relationship. The pictured family tree shows the simplest reconstruction, in which ZWD001 and ZWD002 are interpreted as full brothers.

Unterhautzental

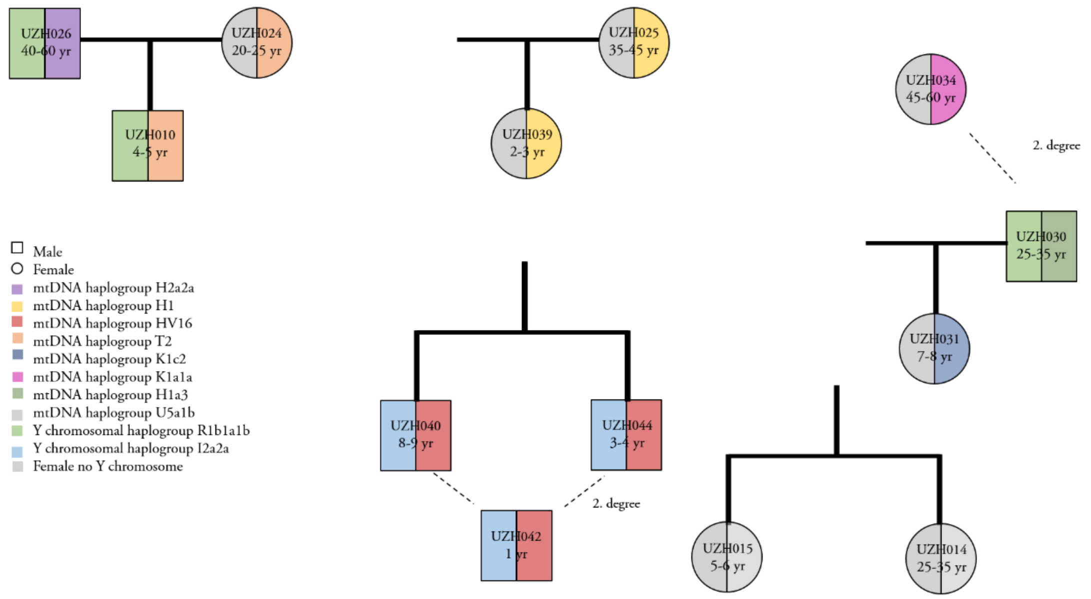

Supplementary Figure 8. Reconstructed family trees Unterhautzental. The pedigree brings together several nuclear families from UZH, comprising multiple adult males and females as well as juveniles. Shared mtDNA and Y-chromosomal haplogroups show that the kin network is linked through both maternal and paternal lines.Genetic distances identify UZH010 as a first-degree relative of both UZH024 and UZH026, most parsimoniously interpreted as their child. UZH014 and UZH015 form another first-degree pair, likely a parent–offspring relationship given the age difference. A further nuclear family centres on UZH030, who is first-degree related to UZH019, UZH031 and UZH034, and second-degree related to UZH020; together these individuals form an extended three-generation cluster. Another close pair is UZH040–UZH044, who are first-degree relatives with UZH042 second-degree related to both, while UZH043 and UZH046 are second-degree relatives forming a separate small unit.The pictured family tree shows the simplest way of arranging these relationships in a pedigree. However, some links are not uniquely determined by the genetic data. For example, the cluster involving UZH019, UZH020, UZH028, UZH030, UZH031 and UZH034 could be realised through different combinations of parent–child, avuncular or cousin relationships; likewise, the second-degree links between UZH014/UZH015 and UZH029 may reflect either a grandparent–grandchild or avuncular/niece–nephew connection. Thus, while the drawn configuration is our most parsimonious interpretation, alternative genealogical realisations of some second-degree ties remain plausible. Furthermore UZH020 is first degree related to UZH031 and second degree related to UZH30. The depicted pedigree represents the most parsimonious arrangement of all first- and second-degree relationships among the individuals shown. Pairwise mismatch rates, however, indicate further close kin from UZH (e.g. the first-degree pair UZH025–UZH039 and several second-degree links involving UZH019, UZH020, UZH028, UZH029, UZH031 and UZH034) that are not included in this figure, and some of the second-degree relationships could also be realised by alternative genealogical configurations.

Schleinbach

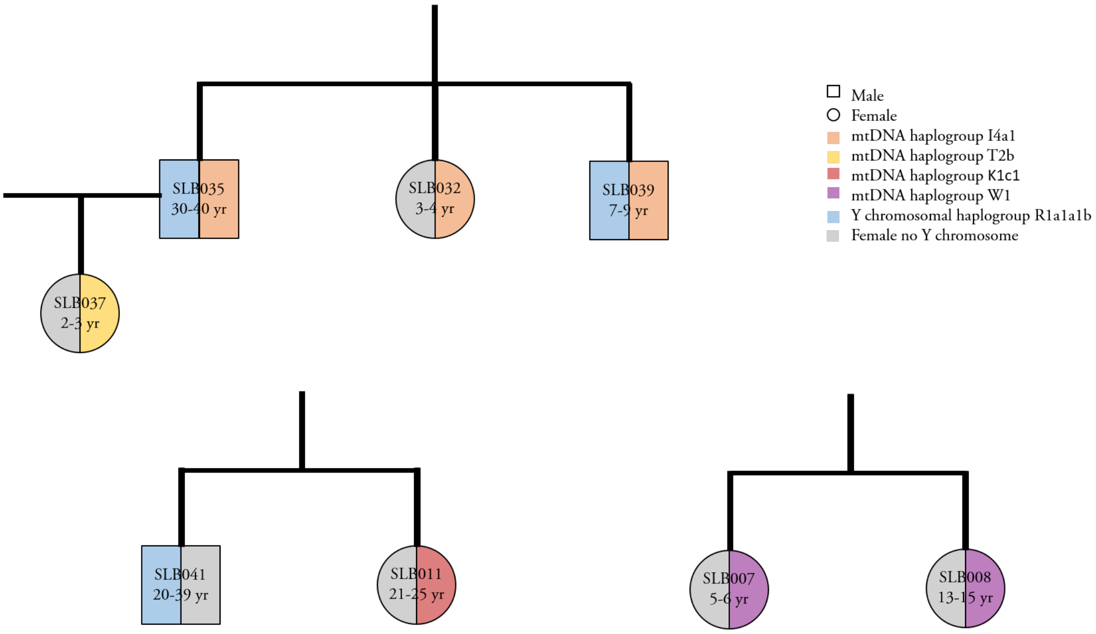

Supplementary Figure 9. Reconstructed family trees Schleinbach. This SLB pedigree comprises eight genetically analysed individuals, four adults (SLB032, SLB035, SLB011, SLB041) and four subadults (SLB007, SLB008, SLB037, SLB039). Based on autosomal pairwise mismatch rates with >1000 overlapping SNPs and shared mtDNA/Y-chromosomal haplogroups, we can reconstruct three nuclear families: a close adult cluster (SLB032, SLB035, SLB039, SLB037) and two pairs of younger individuals (SLB007–SLB008 and SLB011–SLB041). Within the upper cluster, SLB032, SLB035 and SLB039 are all first-degree relatives, and SLB035 is also first-degree related to SLB037, while SLB032–SLB037 and SLB037–SLB039 are second-degree relatives. The drawn pedigree shows the simplest configuration consistent with these data and the osteological ages: SLB032, SLB035 and SLB039 are placed as siblings in one generation, with SLB037 interpreted as the child of SLB035. However, because first-degree kinship alone cannot distinguish unambiguously between parent–child and full siblings, alternative genealogies (e.g. one of the adults being the parent of SLB039 instead of a sibling, or SLB037 as a half-sibling rather than a child) remain plausible but less parsimonious.

For the two lower nuclear families, SLB007 and SLB008 form a first-degree pair, and SLB011 and SLB041 likewise; these are drawn as sibling sets with an unsampled parent in the generation above. Among the individuals depicted, all first- and second-degree relationships with >1000 SNP overlap (SLB007–SLB008, SLB011–SLB041, SLB032–SLB035, SLB032–SLB039, SLB035–SLB037, SLB035–SLB039, SLB032–SLB037, SLB037–SLB039) are represented in the pedigree.

Franzhausen

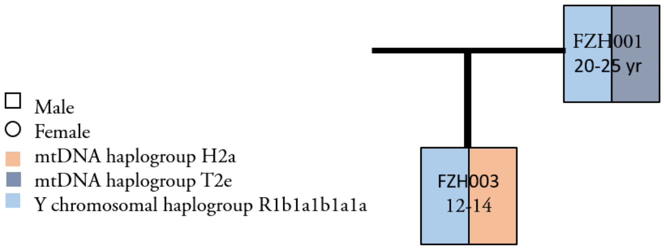

Supplementary Figure 10. Reconstructed family tree Franzhausen. This Franzhausen pedigree comprises two genetically analysed individuals, both males: FZH001, an adult aged about 20–25 years and FZH003, a juvenile aged about 12–14 years. Both carry the same Y-chromosomal haplogroup R1b1a1b1a1a, indicating they share a direct paternal line, while their mtDNA haplogroups differ (H2a vs. T2e), so they do not share a maternal line. Together with their autosomal similarity, this pattern is consistent with a first-degree paternal relationship. In the figure, we therefore reconstruct two consecutive generations, with FZH001 placed in the upper generation and FZH003 in the lower one, linked through the paternal line and an unsampled mother.

Pottenbrunn

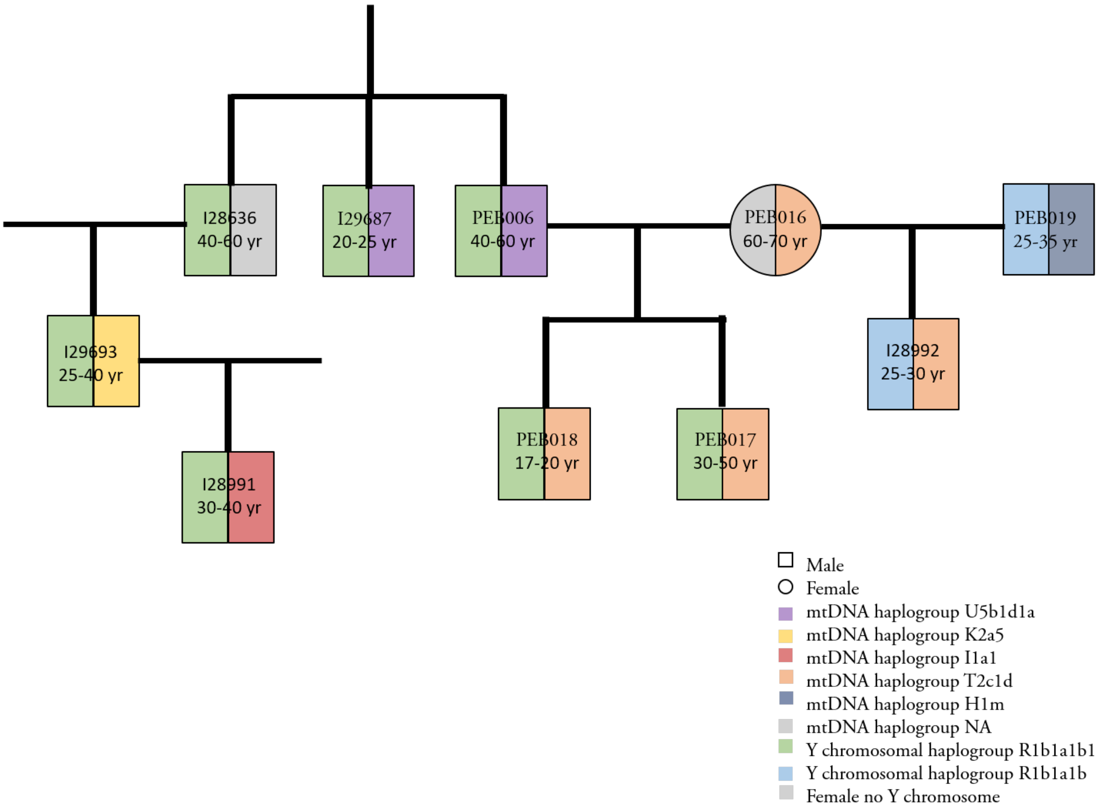

Supplementary Figure 11. Reconstructed family tree 1 Pottenbrunn. This Pottenbrunn pedigree includes ten genetically analysed individuals: nine adults (eight males and one female) and one late-adolescent/subadult male (17–20 years old).

The individuals carry a range of mtDNA haplogroups (U5b1d1a, K2a5, I1a1, T2c1d, H1m and NA) and the males belong to Y-chromosomal haplogroups R1b1a1b1 and R1b1a1b, so the kin network can be followed along both maternal and paternal lines.

The pedigree spans four consecutive generations. At its core are three mutually first-degree related males I28936, I28987 and PEB006, best interpreted, given their similar ages and shared paternal line, as brothers who descend from an unsampled older generation. From I28936 descends a line of males I29993 → I28991, forming a small father–son (or son–father) pair; an unsampled spouse is implied for I29993. PEB006 forms a couple with the older female PEB016 (60–70 yr), and they are shown as the parents of PEB017 (30–50 yr) and the late-adolescent PEB018 (17–20 yr). PEB016 also forms a second union with PEB019 (25–35 yr), from which I28992 (25–30 yr) descends. In this way the pedigree links several male lines through the same maternal ancestor, PEB016.

The drawn family tree represents the simplest configuration that fits the genetic data and osteological ages. Some details, however, are not uniquely determined: for example, the first-degree relationships among I28936, I28987 and PEB006 could in principle represent parent–child rather than sibling ties, and the first-degree links within the pairs I29993–I28991 and PEB017–PEB018 could also be realised as parent–offspring instead of older–younger siblings, given the broad age ranges. Individual PEB005 is possibly first or second-degree related to I28991 & I29693.

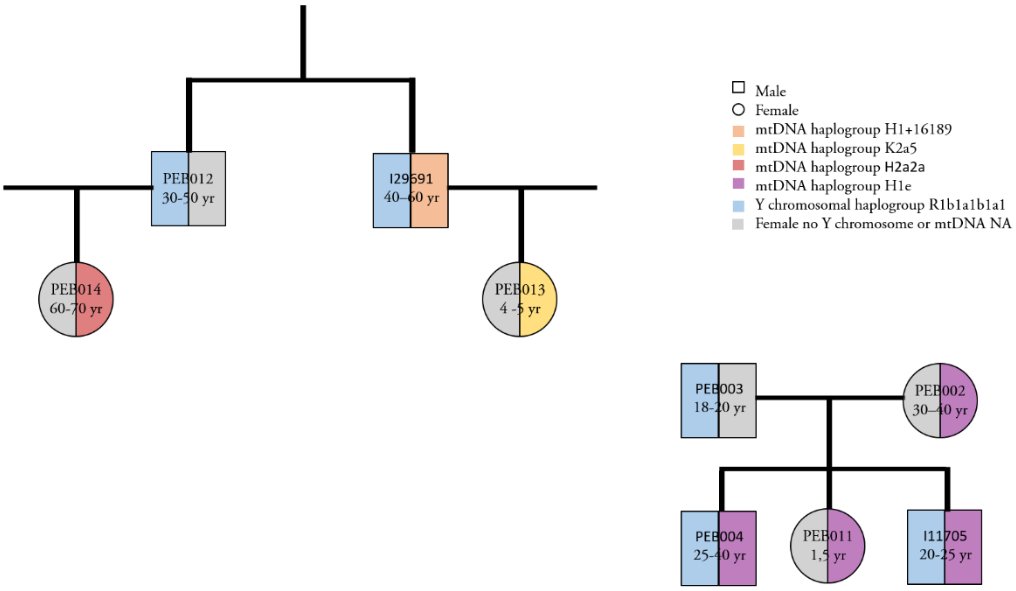

Supplementary Figure 12. Reconstructed family tree 2 Pottenbrunn. This Pottenbrunn figure shows two separate close-kin clusters. The left pedigree comprises four analysed individuals, three adults and one child: Autosomal similarity together with the shared Y haplogroup indicate that PEB012 and I28991 are first-degree relatives on the paternal line, most parsimoniously interpreted as brothers who share a father but have different mothers (explaining their different mtDNA haplogroups). Each of these men is, in turn, a first-degree relative of one younger/older individual: PEB012 with PEB014, and I28991 with PEB013. In the pedigree they are drawn as parent–child pairs, but genetically a first-degree relationship could also represent the reverse generation order or a full-sibling tie. Given the wide and partly overlapping age ranges, the exact genealogical configuration within this small cluster is therefore not uniquely determined; the figure shows the simplest arrangement that fits both the genetic and osteological data.

The right pedigree contains five individuals: All three individuals in the lower row share autosomal first-degree relationships with at least one of the adults above, and their mtDNA and Y-chromosomal haplogroups are consistent with descent from PEB002 and PEB003. The pedigree therefore depicts them as a nuclear family, with PEB002 and PEB003 forming a parental couple and PEB004, PEB011 and I11705 as their children. Because first-degree kinship alone does not distinguish between parent–child and full-siblings, alternative scenarios (for example, one of the “children” being a younger sibling of one of the adults) cannot be completely excluded, especially given the overlapping age estimates. Again, the configuration shown is the most parsimonious interpretation of the data.

Ulrichskirchen

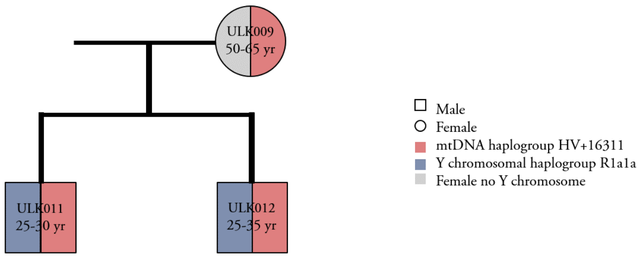

Supplementary Figure 13. Reconstructed family tree Ulrichskirchen. This Ulrichskirchen pedigree consists of three genetically analysed individuals: two young adult males and one older adult female. The two males share both their maternal lineage (same mtDNA haplogroup) and paternal lineage (same Y haplogroup), and each is genetically first-degree related to the older woman. We therefore reconstruct two consecutive generations, with UL009 in the upper generation and UL011 and UL012 in the lower one, linked through both maternal and paternal lines and an unsampled father. In the figure, UL011 and UL012 are interpreted as full brothers, and UL009 as their mother.

The pictured family tree represents the most parsimonious reconstruction that fits the genetic evidence and age estimates. While, in principle, first-degree relationships could also correspond to other configurations (for example, an aunt–nephew relationship), the combination of shared mtDNA and Y-chromosomal haplogroups together with the age ranges makes a mother–sons plus brother–brother scenario by far the most plausible, and no alternative close-kin arrangement among these three individuals is strongly supported.

Supplementary Note 6: Separate IBD networks for males and females

IBD networks were constructed separately for males and females to assess differences in their levels of connectedness. To avoid bias, children for whom parental relationships could be identified in the dataset were included in the analysis. This was done to ensure that the number of connections observed for any individual was not artificially inflated or reduced based on known family ties, which could skew the comparison between males and females. The results show that males generally have more connections than females. Additionally, there are more females without any connections compared to males—five versus two in the Únětice culture and one versus none in the Unterwölbling culture. This pattern further supports the idea of a patrilineal society in these groups.

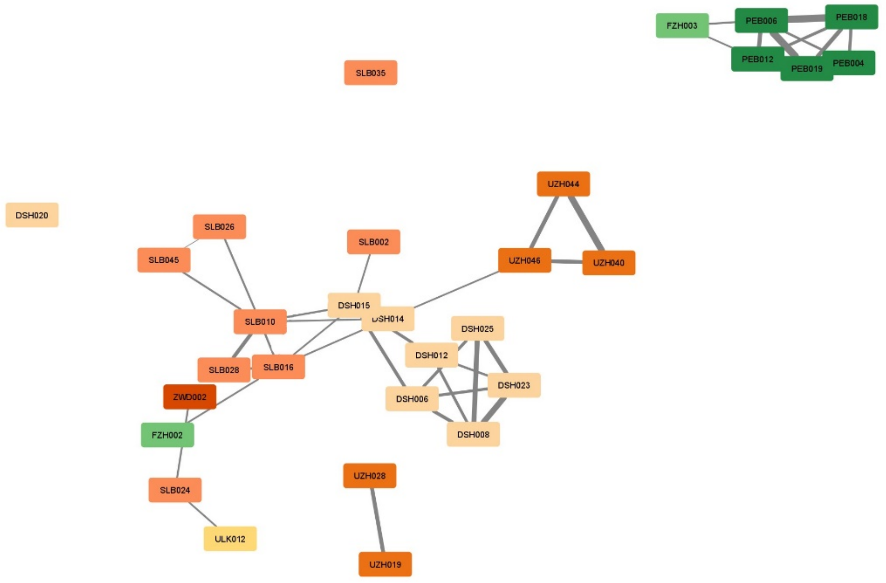

Supplementary Figure 14. IBD network for males in the Únětice (orange) and Unterwölbling (green) cultures. Each square represents an individual male, with different shades indicating the specific site within each culture. The strength of the lines between individuals reflects the maximum IBD (Identity by Descent) value, with thicker lines representing stronger genetic connections.

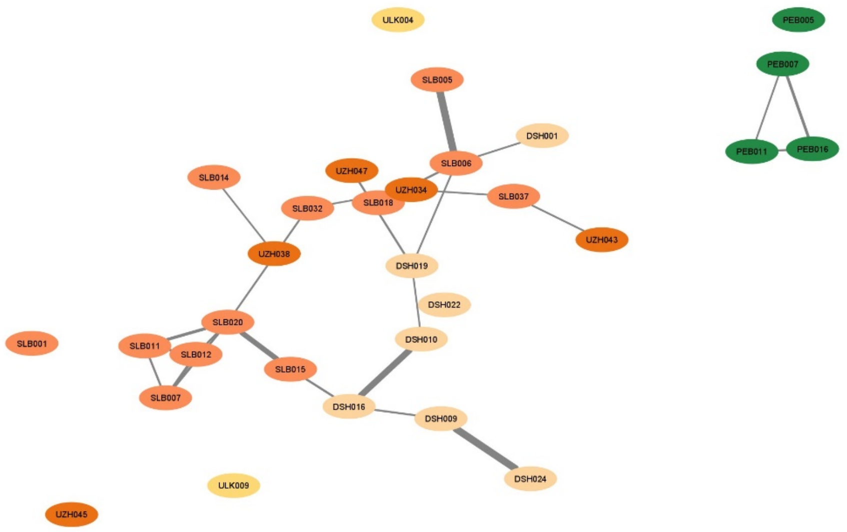

Supplementary Figure 15. IBD network for females in the Únětice (orange) and Unterwölbling (green) cultures. Each circle represents an individual female, with different shades indicating the site within each culture. As with the male network, the strength of the connecting lines indicates the maximum IBD value, with thicker lines showing stronger genetic relationships. The network shows fewer connections among females, with more individuals having no connections, especially in the Únětice culture. This pattern aligns with observations of gender-based differences in social structure and suggests a patrilineal society.

References

1. Fiebig, K. & Csaplaros, A. (eds) *Trassenarchäologie 03* (Archaeoprotect, Pöttelsdorf, 2019).
2. Horváth, T. Das Gräberfeld in Drasenhofen. *in* Fiebig, K. & Csaplaros, A. (eds) *Trassenarchäologie 03, Archpro Sonderheft* **1**, 70–81 (Archaeoprotect, Pöttelsdorf, 2019).
3. Kanz, F. Die frühbronzezeitlichen Menschen in Drasenhofen. *in* Fiebig, K. & Csaplaros, A. (eds) *Trassenarchäologie 03, Archpro Sonderheft* **1**, 82–89 (Archaeoprotect, Pöttelsdorf, 2019).
4. Neumann, G.U. *et al.* Yersinia pestis im frühbronzezeitlichen Gräberfeld von Drasenhofen: die derzeit ältesten Pesttoten Österreichs in ihrem kulturhistorischen Kontext. *Archaeologia Austriaca* **107**, 137–158 (2023).
5. Grefen-Peters, S. Die frühbronzezeitlichen Skelette aus Zwingendorf. Anthropologischer Bericht. *Archaeologia Austriaca* **66**, 49–60 (1982).
6. Wewerka, B. Ein frühbronzezeitliches Gräberfeld aus Zwingendorf, Niederösterreich. *Archaeologia Austriaca* **66**, 21–47 (1982).
7. Lauermann, E. *Ein Frühbronzezeitliches Gräberfeld aus Unterhautzenthal, NÖ* (Amt der Niederösterreichischen Landesregierung, Stockerau, 1995).
8. Lauermann, E. Sonderbestattungen im Bereich einer frühbronzezeitlichen Siedlung in Unterhautzental, Niederösterreich. *in* Rittershofer, K.-F. (ed.) *Sonderbestattungen in der Bronzezeit im östlichen Mitteleuropa, Internationale Archäologie* **37**, 42–46 (Leidorf, Espelkamp, 1997).
9. Lauermann, E., Pucher, E. & Schmitzberger, M. *Unterhautzenthal und Michelberg. Beiträge zum Siedlungswesen der Frühbronzezeitlichen Aunjetitz-Kultur im Nördlichen Niederösterreich. Archäologische Forschungen in Niederösterreich* **1** (NÖ Institut für Landeskunde, St. Pölten, 2001).
10. Rebay-Salisbury, K. *et al.* Tracing mother-child relations in Austrian Early Bronze Age communities through mitochondrial DNA. *in* Meller, H., Krause, J., Haak, W. & Risch, R. (eds) *Kinship, Sex, and Biological Relatedness. The Contribution of Archaeogenetics to the Understanding of Social and Biological Relations, Proceedings of the 15th Archaeological Conference of Central Germany*, 213–226 (Landesmuseum für Vorgeschichte, Halle/Saale, 2023).
11. Rebay-Salisbury, K. *et al.* Motherhood at early Bronze Age Unterhautzenthal, Lower Austria. *Archaeologia Austriaca* **102**, 71–134 (2018).
12. Rebay-Salisbury, K. *et al.* Child murder in the Early Bronze Age: proteomic sex identification of a cold case from Schleinbach, Austria. *Archaeol. Anthropol. Sci.* **12**, 265 (2020).
13. Rettenbacher, M. *Die Siedlung und die Gräberfelder von Schleinbach: Eine Studie zur Aunjetitz-Kultur im Südlichen Weinviertel. Archäologische Forschungen in Niederösterreich* **2** (Niederösterreichisches Institut für Landeskunde, St. Pölten, 2004).
14. Weninger, M. Frühbronzezeitliche Skelette aus Schleinbach in Niederösterreich. Teil 2: die Einzelbestattungen aus Schleinbach (NÖ). *Archaeologia Austriaca* **16**, 28–66 (1954).

15. Pany-Kucera, D. *et al.* Social Relations, Deprivation and Violence at Schleinbach, Lower Austria: Insights from an interdisciplinary analysis of the Early Bronze Age human remains. *Archaeologia Austriaca* **104**, 13–52 (2020).

16. Verdianu, D. *Die Frühbronzezeitlichen Gräber aus Ulrichskirchen: Gräberfeld und Sonderbestattungen im Vergleich* (Universität Wien, Vienna, 2024).

17. Neugebauer, J.-W. (ed.) *Bronzezeit in Ostösterreich. Wissenschaftliche Schriftenreihe Niederösterreich* **98–101** (Niederösterreichisches Pressehaus, Wien-St. Pölten, 1994).

18. Neugebauer, C. & Neugebauer, J.-W. *Franzhausen: Das Frühbronzezeitliche Gräberfeld I. Fundberichte Österreich Materialhefte Reihe A* **5/1 und 2** (Berger, Horn, 1997).

19. Rebay-Salisbury, K. Personal Relationships between Co-buried Individuals in the Central European Early Bronze Age. *in* Lillehammer, G. & Murphy, E. (eds) *Across the Generations: The Old and the Young in Past Societies, Childhood in the Past Monograph Series* **8**, 35–48 (Museum of Archaeology/University of Stavanger, Stavanger, 2018).

20. Blesl, C. *Das Frühbronzezeitliche Gräberfeld von Pottenbrunn. Fundberichte aus Österreich, Materialheft A* **15** (Berger, Horn, 2006).

21. Novotny, F. Die krankhaften und traumatischen Veränderungen an den frühbronzezeitlichen Skelettresten des Gräberfeldes Pottenbrunn. *in* Blesl, C. (ed.) *Das Frühbronzezeitliche Gräberfeld von Pottenbrunn, Fundberichte aus Österreich, Materialheft A* **15**, 147–202 (Berger, Horn, 2006).

22. Ramsl, P.C. *Das Eisenzeitliche Gräberfeld von Pottenbrunn. Fundberichte aus Österreich, Materialhefte A* **11** (Berger, Horn, 2002).
