# Supplementary material for: Tracing social mechanisms and interregional connections in Early Bronze Age Societies in Lower Austria
Source: Nat Commun. 2025 Dec 31;17:131. doi: 10.1038/s41467-025-67906-y (PMC12775072; doi:10.1038/s41467-025-67906-y)
Supplement: Supplementary file 2 — Description of Additional Supplementary Files [file 41467_2025_67906_MOESM2_ESM.pdf]

Title: Supplementary Data 1

Description: Sequencing and quality control metrics for each sample, including read counts, endogenous DNA percentages, damage patterns, mitochondrial and Y-chromosome haplogroups, and radiocarbon dating information with calibrated date ranges and laboratory identifiers.

Title: Supplementary Data 2

Description: Library data from individuals processed in the Reich Laboratory, including laboratory identifiers, quality assessments, archaeological metadata, radiocarbon dates, sequencing statistics, mtDNA damage rates, haplogroups, 1240K SNP coverage, and genetically inferred sex.

Title: Supplementary Data 3

Description: Ancestry estimates for each sample showing proportions of Western Hunter-Gatherer (WHG), Anatolian Neolithic, and Yamnaya-related ancestry with standard errors, cultural assignments, and archaeological context metadata.

Title: Supplementary Data 4

Description: Pairwise genetic relatedness data including mismatch rates, overlapping SNP counts, kinship coefficients, and X chromosome-specific statistics for assessing biological relationships. Relationship estimates integrate results from multiple methods (PWM, READ, NGSrelate) for enhanced reliability.

Title: Supplementary Data 5

Description: Pairwise identity-by-descent (IBD) sharing data including maximum IBD segment lengths and total shared segments above various length thresholds, providing insights into recent genetic relatedness within and between archaeological sites.

Title: Supplementary Data 6

Description: Genotypes at selected SNPs of interest (including lactase persistence markers) across all individuals, with allelic read counts formatted as reference/alternate.

Title: Supplementary Data 7

Description: Archaeological and genetic metadata summary for each sample including site location, grave number, archaeological identifiers, estimated age, genetic sex, and screening outcomes, serving as a contextual reference for analyzed individuals.
